# 2-{[5-(Adamantan-1-yl)-4-methyl-4*H*-1,2,4-triazol-3-yl]sulfan­yl}-*N*,*N*-dimethyl­ethanamine

**DOI:** 10.1107/S160053681201464X

**Published:** 2012-04-13

**Authors:** Ali A. El-Emam, Siham Lahsasni, Hanadi H. Asiri, Ching Kheng Quah, Hoong-Kun Fun

**Affiliations:** a Department of Pharmaceutical Chemistry, College of Pharmacy, King Saud University, Riyadh 11451, Saudi Arabia; b Department of Chemistry, College of Sciences, King Saud University, Riyadh, Saudi Arabia; c X-ray Crystallography Unit, School of Physics, Universiti Sains Malaysia, 11800 USM, Penang, Malaysia

## Abstract

In the title compound, C_17_H_28_N_4_S, the 1,2,4-triazole ring is nearly planar [maximum deviation = 0.005 (2) Å]. There are no significant hydrogen bonds observed in the crystal structure. The crystal studied was a non-merohedral twin, the refined ratio of twin components being 0.281 (3):0.719 (3).

## Related literature

For the biological activity of adamantyl derivatives see: Al-Omar *et al.* (2010 ▶); Al-Deeb *et al.* (2006 ▶); El-Emam *et al.* (2004 ▶); Kadi *et al.* (2007 ▶, 2010 ▶); Vernier *et al.* (1969 ▶). For the structures of related adamantyl-1,2,4-triazoles, see: Almutairi *et al.* (2012 ▶); Al-Tamimi *et al.* (2010 ▶); Al-Abdullah *et al.* (2012 ▶). For the structures of substituted sulfanyl-1,2,4-triazoles, see: Fun *et al.* (2011 ▶); Wang *et al.* (2011 ▶). For standard bond-length data, see: Allen *et al.* (1987 ▶).
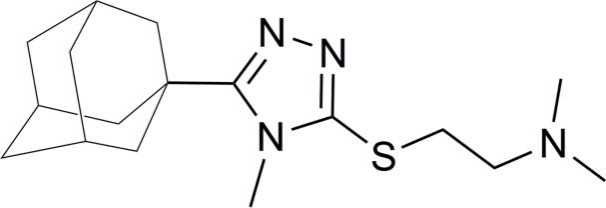


## Experimental

### 

#### Crystal data

C_17_H_28_N_4_S
*M* *_r_* = 320.49
Monoclinic, 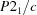

*a* = 12.5133 (7) Å
*b* = 10.3779 (5) Å
*c* = 14.3044 (8) Å
β = 106.766 (3)°
*V* = 1778.63 (16) Å^3^
*Z* = 4
Cu *K*α radiation
μ = 1.62 mm^−1^
*T* = 296 K
0.64 × 0.59 × 0.05 mm

#### Data collection

Bruker SMART APEXII CCD area-detector diffractometer
Absorption correction: multi-scan (*SADABS*; Bruker, 2009 ▶) *T*_min_ = 0.204, *T*_max_ = 0.923
3267 measured reflections
3267 independent reflections
2846 reflections with *I* > 2σ(*I*)

#### Refinement

*R*[*F* ^2^ > 2σ(*F* ^2^)] = 0.064
*wR*(*F* ^2^) = 0.184
*S* = 1.13
3267 reflections
203 parameters
H-atom parameters constrained
Δρ_max_ = 0.32 e Å^−3^
Δρ_min_ = −0.37 e Å^−3^

### 

Data collection: *APEX2* (Bruker, 2009 ▶); cell refinement: *SAINT* (Bruker, 2009 ▶); data reduction: *SAINT*; program(s) used to solve structure: *SHELXTL* (Sheldrick, 2008 ▶); program(s) used to refine structure: *SHELXTL*; molecular graphics: *SHELXTL*; software used to prepare material for publication: *SHELXTL* and *PLATON* (Spek, 2009 ▶).

## Supplementary Material

Crystal structure: contains datablock(s) global, I. DOI: 10.1107/S160053681201464X/rz2734sup1.cif

Structure factors: contains datablock(s) I. DOI: 10.1107/S160053681201464X/rz2734Isup2.hkl

Supplementary material file. DOI: 10.1107/S160053681201464X/rz2734Isup3.cml

Additional supplementary materials:  crystallographic information; 3D view; checkCIF report

## Figures and Tables

**Fig. 1.**
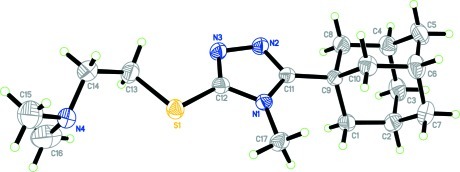
The molecular structure of the title compound showing 30% probability displacement ellipsoids for non-H atoms.

## supplementary crystallographic information

### Comment

Considerable attention has been devoted to adamantane derivatives which have long been known for their diverse biological properties as antiviral against the influenza (Vernier *et al.*, 1969) and HIV viruses (El-Emam, Al-Deeb, Al-Omar & Lehmann, 2004). Moreover, adamantane derivatives were recently reported to exhibit marked antibacterial activity (Kadi *et al.*, 2007, 2010). In continuation of our interest in the chemical and pharmacological properties of adamantane derivatives, we synthesized the title compound as a potential chemotherapeutic agent.

In the title molecule, Fig. 1, the 1,2,4-triazole ring (N1-N3/C11/C12) is nearly planar with a maximum deviation of 0.005 (2) Å at atom N2. Bond lengths (Allen *et al.*, 1987) and angles are within normal ranges and are comparable to those reported for related structures (Almutairi *et al.*, 2012; Al-Tamimi *et al.*, 2010; Al-Abdullah *et al.*, 2012; Fun *et al.*, 2011; Wang *et al.*, 2011). The crystal studied was a non-merohedral twin, the refined ratio of twin components being 0.281 (3):0.719 (3). There are no significant hydrogen bonds observed in this compound.

### Experimental

A mixture of 3-(adamantan-1-yl)-4-methyl-4*H*-1,2,4-triazole-5-thiol (2.49 g, 0.01 mol), potassium hydroxide (1.12 g, 0.02 mol) and 2-dimethylaminoethyl chloride hydrochloride (1.44 g, 0.01 mol) in ethanol (15 ml) was heated under reflux with stirring for 3 h and the solvent was distilled off *in vacuo*. The obtained residue was washed with water and purified by column chromatography on silica gel column using CHCl_3_:MeOH (9:1 *v*/*v*) as eluent to yield 2.02 g (63%) of the title compound as colorless powder. M.p. 133-135°C. Single crystals suitable for X-ray diffraction were obtained by crystallization from aqueous ethanol. ^1^H NMR (CDCl_3_, 500.13 MHz): δ 1.69-1.75 (m, 6H, adamantane-H), 2.04-2.85 (m, 9H, adamantane-H), 2.21 (s, 6H, 2xCH_3_), 2.62 (t, 2H, CH_2_N, *J* = 6.5 Hz), 3.28 (t, 2H, SCH_2_, *J* = 6.5 Hz), 3.59 (s, 3H, CH_3_). ^13^C NMR (CDCl_3_, 125.76 MHz): δ 28.07, 34.98, 36.50, 49.56 (adamantane-C), 31.05 (CH_3_), 32.33 (SCH_2_), 45.21 (2xCH_3_), 58.24 (CH_2_N), 152.16, 161.21 (triazole C).

### Refinement

All hydrogen atoms were positioned geometrically [C–H = 0.96–0.98 Å] and refined using a riding model, with *U*_iso_(H) = 1.2 or 1.5 *U*_eq_(C). A rotating group model was applied to the methyl groups. The crystal studied was a non-merohedral twin, the refined ratio of twin components being 0.281 (3):0.719 (3).

### Crystal data

**Table:**

C_17_H_28_N_4_S	*F*(000) = 696
*M**_r_* = 320.49	*D*_x_ = 1.197 Mg m^−^^3^
Monoclinic, *P*2_1_/*c*	Cu *K*α radiation, λ = 1.54178 Å
Hall symbol: -P 2ybc	Cell parameters from 3932 reflections
*a* = 12.5133 (7) Å	θ = 7.7–69.2°
*b* = 10.3779 (5) Å	µ = 1.62 mm^−^^1^
*c* = 14.3044 (8) Å	*T* = 296 K
β = 106.766 (3)°	Plate, colourless
*V* = 1778.63 (16) Å^3^	0.64 × 0.59 × 0.05 mm
*Z* = 4	

### Data collection

**Table:**

Bruker SMART APEXII CCD area-detector diffractometer	3267 independent reflections
Radiation source: fine-focus sealed tube	2846 reflections with *I* > 2σ(*I*)
Graphite monochromator	*R*_int_ = 0.000
φ and ω scans	θ_max_ = 69.8°, θ_min_ = 7.7°
Absorption correction: multi-scan (*SADABS*; Bruker, 2009)	*h* = −15→14
*T*_min_ = 0.204, *T*_max_ = 0.923	*k* = −12→12
3267 measured reflections	*l* = 0→16

### Refinement

**Table:**

Refinement on *F*^2^	Primary atom site location: structure-invariant direct methods
Least-squares matrix: full	Secondary atom site location: difference Fourier map
*R*[*F*^2^ > 2σ(*F*^2^)] = 0.064	Hydrogen site location: inferred from neighbouring sites
*wR*(*F*^2^) = 0.184	H-atom parameters constrained
*S* = 1.13	*w* = 1/[σ^2^(*F*_o_^2^) + (0.*P*)^2^ + 1.1111*P*] where *P* = (*F*_o_^2^ + 2*F*_c_^2^)/3
3267 reflections	(Δ/σ)_max_ = 0.001
203 parameters	Δρ_max_ = 0.32 e Å^−^^3^
0 restraints	Δρ_min_ = −0.37 e Å^−^^3^

### Special details

**Table:**

Geometry. All esds (except the esd in the dihedral angle between two l.s. planes) are estimated using the full covariance matrix. The cell esds are taken into account individually in the estimation of esds in distances, angles and torsion angles; correlations between esds in cell parameters are only used when they are defined by crystal symmetry. An approximate (isotropic) treatment of cell esds is used for estimating esds involving l.s. planes.
Refinement. Refinement of F^2^ against ALL reflections. The weighted R-factor wR and goodness of fit S are based on F^2^, conventional R-factors R are based on F, with F set to zero for negative F^2^. The threshold expression of F^2^ > 2sigma(F^2^) is used only for calculating R-factors(gt) etc. and is not relevant to the choice of reflections for refinement. R-factors based on F^2^ are statistically about twice as large as those based on F, and R- factors based on ALL data will be even larger.

### Fractional atomic coordinates and isotropic or equivalent isotropic displacement parameters (Å^2^)

**Table:**

	*x*	*y*	*z*	*U*_iso_*/*U*_eq_	
S1	0.59451 (7)	0.30472 (8)	0.05471 (7)	0.0589 (3)	
N1	0.4306 (2)	0.2967 (2)	0.14471 (19)	0.0478 (6)	
N4	0.7649 (3)	0.2967 (3)	−0.0644 (2)	0.0630 (7)	
C9	0.2838 (2)	0.2248 (3)	0.2293 (2)	0.0476 (7)	
C10	0.3344 (3)	0.2856 (4)	0.3307 (3)	0.0672 (9)	
H10A	0.3944	0.2315	0.3689	0.081*	
H10B	0.3653	0.3694	0.3234	0.081*	
C6	0.2449 (4)	0.3001 (5)	0.3838 (3)	0.0814 (12)	
H6A	0.2779	0.3402	0.4477	0.098*	
C5	0.1995 (4)	0.1692 (5)	0.3976 (3)	0.0893 (14)	
H5A	0.1441	0.1778	0.4328	0.107*	
H5B	0.2594	0.1149	0.4359	0.107*	
C4	0.1465 (4)	0.1071 (4)	0.2985 (3)	0.0765 (11)	
H4A	0.1169	0.0222	0.3077	0.092*	
C3	0.0526 (3)	0.1919 (5)	0.2391 (3)	0.0817 (13)	
H3A	0.0183	0.1524	0.1761	0.098*	
H3B	−0.0041	0.2011	0.2728	0.098*	
C13	0.6508 (4)	0.1620 (4)	0.0134 (4)	0.0803 (12)	
H13A	0.6560	0.0927	0.0600	0.096*	
H13B	0.6015	0.1344	−0.0490	0.096*	
C2	0.0985 (3)	0.3229 (4)	0.2249 (3)	0.0757 (11)	
H2A	0.0375	0.3775	0.1869	0.091*	
C7	0.1513 (4)	0.3853 (5)	0.3230 (4)	0.0861 (13)	
H7A	0.0954	0.3977	0.3571	0.103*	
H7B	0.1810	0.4691	0.3136	0.103*	
C1	0.1858 (3)	0.3084 (4)	0.1699 (3)	0.0653 (9)	
H1A	0.2132	0.3927	0.1586	0.078*	
H1B	0.1520	0.2684	0.1069	0.078*	
C8	0.2350 (3)	0.0923 (3)	0.2444 (3)	0.0666 (9)	
H8A	0.2018	0.0521	0.1814	0.080*	
H8B	0.2944	0.0367	0.2817	0.080*	
C11	0.3702 (2)	0.2040 (3)	0.1772 (2)	0.0477 (7)	
N2	0.4021 (2)	0.0897 (2)	0.1572 (2)	0.0577 (7)	
N3	0.4859 (3)	0.1046 (3)	0.1120 (2)	0.0599 (7)	
C12	0.5001 (3)	0.2289 (3)	0.1054 (2)	0.0516 (7)	
C14	0.7641 (4)	0.1909 (5)	0.0031 (4)	0.0874 (14)	
H14A	0.8139	0.2124	0.0669	0.105*	
H14B	0.7930	0.1140	−0.0196	0.105*	
C16	0.7117 (7)	0.2636 (6)	−0.1633 (5)	0.126 (2)	
H16A	0.7180	0.3341	−0.2048	0.189*	
H16B	0.6342	0.2457	−0.1713	0.189*	
H16C	0.7468	0.1886	−0.1805	0.189*	
C17	0.4269 (3)	0.4366 (3)	0.1495 (3)	0.0633 (9)	
H17A	0.4899	0.4723	0.1330	0.095*	
H17B	0.4292	0.4626	0.2145	0.095*	
H17C	0.3593	0.4674	0.1043	0.095*	
C15	0.8793 (5)	0.3349 (7)	−0.0500 (6)	0.120 (2)	
H15A	0.8822	0.4023	−0.0951	0.181*	
H15B	0.9217	0.2623	−0.0608	0.181*	
H15C	0.9101	0.3655	0.0156	0.181*	

### Atomic displacement parameters (Å^2^)

**Table:**

	*U*^11^	*U*^22^	*U*^33^	*U*^12^	*U*^13^	*U*^23^
S1	0.0605 (5)	0.0553 (5)	0.0668 (6)	−0.0051 (3)	0.0278 (4)	−0.0023 (4)
N1	0.0524 (14)	0.0418 (13)	0.0497 (15)	0.0000 (10)	0.0156 (11)	0.0003 (10)
N4	0.0598 (17)	0.0665 (18)	0.0661 (19)	0.0050 (13)	0.0236 (14)	0.0030 (14)
C9	0.0478 (15)	0.0505 (16)	0.0430 (16)	0.0012 (12)	0.0105 (13)	−0.0010 (12)
C10	0.060 (2)	0.084 (2)	0.051 (2)	−0.0009 (18)	0.0042 (16)	−0.0097 (17)
C6	0.076 (3)	0.113 (4)	0.054 (2)	−0.003 (2)	0.0162 (19)	−0.021 (2)
C5	0.082 (3)	0.133 (4)	0.059 (3)	0.012 (3)	0.031 (2)	0.017 (2)
C4	0.079 (3)	0.078 (3)	0.083 (3)	−0.011 (2)	0.039 (2)	0.003 (2)
C3	0.056 (2)	0.123 (4)	0.070 (3)	−0.011 (2)	0.0232 (19)	−0.009 (2)
C13	0.081 (3)	0.064 (2)	0.114 (4)	0.0078 (19)	0.056 (3)	0.006 (2)
C2	0.057 (2)	0.095 (3)	0.073 (3)	0.0195 (19)	0.0154 (18)	0.007 (2)
C7	0.085 (3)	0.092 (3)	0.091 (3)	0.009 (2)	0.042 (3)	−0.018 (2)
C1	0.059 (2)	0.080 (2)	0.055 (2)	0.0125 (16)	0.0139 (16)	0.0096 (17)
C8	0.070 (2)	0.063 (2)	0.072 (2)	−0.0051 (16)	0.0289 (18)	0.0026 (17)
C11	0.0505 (16)	0.0440 (15)	0.0476 (17)	0.0002 (12)	0.0126 (13)	0.0023 (12)
N2	0.0657 (16)	0.0454 (14)	0.0686 (18)	0.0029 (12)	0.0299 (14)	0.0032 (12)
N3	0.0682 (17)	0.0466 (15)	0.0727 (19)	0.0041 (12)	0.0327 (15)	0.0027 (12)
C12	0.0531 (17)	0.0499 (17)	0.0499 (18)	0.0020 (13)	0.0118 (14)	0.0008 (13)
C14	0.080 (3)	0.094 (3)	0.096 (3)	0.024 (2)	0.038 (3)	0.024 (3)
C16	0.181 (6)	0.097 (4)	0.082 (4)	0.030 (4)	0.008 (4)	−0.013 (3)
C17	0.069 (2)	0.0442 (17)	0.080 (2)	−0.0017 (15)	0.0273 (18)	−0.0028 (15)
C15	0.082 (3)	0.137 (5)	0.151 (6)	0.003 (3)	0.047 (4)	0.032 (4)

### Geometric parameters (Å, º)

**Table:**

S1—C12	1.742 (3)	C13—C14	1.498 (6)
S1—C13	1.811 (4)	C13—H13A	0.9700
N1—C12	1.361 (4)	C13—H13B	0.9700
N1—C11	1.384 (4)	C2—C7	1.513 (7)
N1—C17	1.455 (4)	C2—C1	1.527 (5)
N4—C16	1.421 (7)	C2—H2A	0.9800
N4—C15	1.441 (6)	C7—H7A	0.9700
N4—C14	1.464 (5)	C7—H7B	0.9700
C9—C11	1.495 (4)	C1—H1A	0.9700
C9—C10	1.539 (5)	C1—H1B	0.9700
C9—C1	1.542 (4)	C8—H8A	0.9700
C9—C8	1.546 (5)	C8—H8B	0.9700
C10—C6	1.531 (6)	C11—N2	1.310 (4)
C10—H10A	0.9700	N2—N3	1.390 (4)
C10—H10B	0.9700	N3—C12	1.309 (4)
C6—C5	1.508 (7)	C14—H14A	0.9700
C6—C7	1.524 (7)	C14—H14B	0.9700
C6—H6A	0.9800	C16—H16A	0.9600
C5—C4	1.524 (7)	C16—H16B	0.9600
C5—H5A	0.9700	C16—H16C	0.9600
C5—H5B	0.9700	C17—H17A	0.9600
C4—C3	1.517 (7)	C17—H17B	0.9600
C4—C8	1.531 (5)	C17—H17C	0.9600
C4—H4A	0.9800	C15—H15A	0.9600
C3—C2	1.513 (7)	C15—H15B	0.9600
C3—H3A	0.9700	C15—H15C	0.9600
C3—H3B	0.9700		
			
C12—S1—C13	98.05 (17)	C7—C2—H2A	109.2
C12—N1—C11	104.8 (2)	C1—C2—H2A	109.2
C12—N1—C17	124.6 (3)	C2—C7—C6	109.8 (4)
C11—N1—C17	130.5 (3)	C2—C7—H7A	109.7
C16—N4—C15	111.7 (5)	C6—C7—H7A	109.7
C16—N4—C14	112.6 (4)	C2—C7—H7B	109.7
C15—N4—C14	107.9 (4)	C6—C7—H7B	109.7
C11—C9—C10	111.7 (3)	H7A—C7—H7B	108.2
C11—C9—C1	112.4 (3)	C2—C1—C9	110.2 (3)
C10—C9—C1	109.5 (3)	C2—C1—H1A	109.6
C11—C9—C8	108.2 (3)	C9—C1—H1A	109.6
C10—C9—C8	107.7 (3)	C2—C1—H1B	109.6
C1—C9—C8	107.1 (3)	C9—C1—H1B	109.6
C6—C10—C9	110.3 (3)	H1A—C1—H1B	108.1
C6—C10—H10A	109.6	C4—C8—C9	110.7 (3)
C9—C10—H10A	109.6	C4—C8—H8A	109.5
C6—C10—H10B	109.6	C9—C8—H8A	109.5
C9—C10—H10B	109.6	C4—C8—H8B	109.5
H10A—C10—H10B	108.1	C9—C8—H8B	109.5
C5—C6—C7	109.9 (4)	H8A—C8—H8B	108.1
C5—C6—C10	109.5 (4)	N2—C11—N1	109.0 (3)
C7—C6—C10	109.0 (4)	N2—C11—C9	123.3 (3)
C5—C6—H6A	109.5	N1—C11—C9	127.6 (3)
C7—C6—H6A	109.5	C11—N2—N3	108.6 (3)
C10—C6—H6A	109.5	C12—N3—N2	106.3 (3)
C6—C5—C4	109.8 (3)	N3—C12—N1	111.2 (3)
C6—C5—H5A	109.7	N3—C12—S1	126.8 (3)
C4—C5—H5A	109.7	N1—C12—S1	122.0 (2)
C6—C5—H5B	109.7	N4—C14—C13	113.7 (4)
C4—C5—H5B	109.7	N4—C14—H14A	108.8
H5A—C5—H5B	108.2	C13—C14—H14A	108.8
C3—C4—C5	109.5 (4)	N4—C14—H14B	108.8
C3—C4—C8	109.4 (4)	C13—C14—H14B	108.8
C5—C4—C8	109.2 (4)	H14A—C14—H14B	107.7
C3—C4—H4A	109.6	N4—C16—H16A	109.5
C5—C4—H4A	109.6	N4—C16—H16B	109.5
C8—C4—H4A	109.6	H16A—C16—H16B	109.5
C2—C3—C4	109.5 (3)	N4—C16—H16C	109.5
C2—C3—H3A	109.8	H16A—C16—H16C	109.5
C4—C3—H3A	109.8	H16B—C16—H16C	109.5
C2—C3—H3B	109.8	N1—C17—H17A	109.5
C4—C3—H3B	109.8	N1—C17—H17B	109.5
H3A—C3—H3B	108.2	H17A—C17—H17B	109.5
C14—C13—S1	109.7 (3)	N1—C17—H17C	109.5
C14—C13—H13A	109.7	H17A—C17—H17C	109.5
S1—C13—H13A	109.7	H17B—C17—H17C	109.5
C14—C13—H13B	109.7	N4—C15—H15A	109.5
S1—C13—H13B	109.7	N4—C15—H15B	109.5
H13A—C13—H13B	108.2	H15A—C15—H15B	109.5
C3—C2—C7	110.0 (4)	N4—C15—H15C	109.5
C3—C2—C1	109.7 (4)	H15A—C15—H15C	109.5
C7—C2—C1	109.6 (3)	H15B—C15—H15C	109.5
C3—C2—H2A	109.2		
			
C11—C9—C10—C6	−177.5 (3)	C10—C9—C8—C4	58.6 (4)
C1—C9—C10—C6	57.3 (4)	C1—C9—C8—C4	−59.2 (4)
C8—C9—C10—C6	−58.8 (4)	C12—N1—C11—N2	−0.5 (4)
C9—C10—C6—C5	60.9 (4)	C17—N1—C11—N2	−179.8 (3)
C9—C10—C6—C7	−59.3 (5)	C12—N1—C11—C9	178.0 (3)
C7—C6—C5—C4	58.9 (5)	C17—N1—C11—C9	−1.3 (5)
C10—C6—C5—C4	−60.8 (5)	C10—C9—C11—N2	113.2 (4)
C6—C5—C4—C3	−59.6 (5)	C1—C9—C11—N2	−123.3 (4)
C6—C5—C4—C8	60.2 (5)	C8—C9—C11—N2	−5.3 (4)
C5—C4—C3—C2	59.8 (5)	C10—C9—C11—N1	−65.1 (4)
C8—C4—C3—C2	−59.8 (5)	C1—C9—C11—N1	58.4 (4)
C12—S1—C13—C14	−157.1 (3)	C8—C9—C11—N1	176.4 (3)
C4—C3—C2—C7	−59.9 (5)	N1—C11—N2—N3	0.8 (4)
C4—C3—C2—C1	60.7 (4)	C9—C11—N2—N3	−177.8 (3)
C3—C2—C7—C6	59.1 (5)	C11—N2—N3—C12	−0.8 (4)
C1—C2—C7—C6	−61.6 (5)	N2—N3—C12—N1	0.5 (4)
C5—C6—C7—C2	−58.6 (5)	N2—N3—C12—S1	−179.9 (3)
C10—C6—C7—C2	61.3 (5)	C11—N1—C12—N3	−0.1 (4)
C3—C2—C1—C9	−61.4 (4)	C17—N1—C12—N3	179.3 (3)
C7—C2—C1—C9	59.4 (5)	C11—N1—C12—S1	−179.7 (2)
C11—C9—C1—C2	178.2 (3)	C17—N1—C12—S1	−0.3 (5)
C10—C9—C1—C2	−57.1 (4)	C13—S1—C12—N3	3.1 (4)
C8—C9—C1—C2	59.5 (4)	C13—S1—C12—N1	−177.3 (3)
C3—C4—C8—C9	60.2 (5)	C16—N4—C14—C13	−69.9 (6)
C5—C4—C8—C9	−59.7 (5)	C15—N4—C14—C13	166.4 (5)
C11—C9—C8—C4	179.4 (3)	S1—C13—C14—N4	−58.1 (5)
